# Ahead of the game protocol: a multi-component, community sport-based program targeting prevention, promotion and early intervention for mental health among adolescent males

**DOI:** 10.1186/s12889-018-5319-7

**Published:** 2018-03-21

**Authors:** Stewart A. Vella, Christian Swann, Marijka Batterham, Katherine M. Boydell, Simon Eckermann, Andrea Fogarty, Diarmuid Hurley, Sarah K. Liddle, Chris Lonsdale, Andrew Miller, Michael Noetel, Anthony D. Okely, Taren Sanders, Joanne Telenta, Frank P. Deane

**Affiliations:** 1 School of Psychology and Early Start, Faculty of Social Sciences, University of Wollongong, Northfields Avenue, Wollongong, 2522 Australia; 2 School of Mathematics and Applied Statistics, Faculty of Engineering and Information Sciences, University of Wollongong, Northfields Avenue, Wollongong, 2522 Australia; 3 Black Dog Institute, University of New South Wales, Hospital Road, Randwick, 2031 Australia; 4 Australian Health Services Research Institute, Sydney Business School, Faculty of Business, University of Wollongong, Northfields Avenue, Wollongong, 2522 Australia; 5 School of Psychology, Faculty of Social Sciences, University of Wollongong, Northfields Avenue, Wollongong, 2522 Australia; 6 Institute for Positive Psychology and Education, Faculty of Health Sciences, Australian Catholic University, 25A Barker Road, Strathfield, 2135 Australia; 7 School of Education, University of Newcastle, EN2.05 Chittaway Road, Ourimbah, Ourimbah, 2258 Australia; 8 School of Exercise Science, Faculty of Health Sciences, Australian Catholic University, 25A Barker Road, Strathfield, 2135 Australia; 9 Early Start, Faculty of Social Sciences, University of Wollongong, Northfields Avenue, Wollongong, 2522 Australia; 10 Centre for Health and Social Research, St. Patrick’s Campus, Level 5, 215 Spring St, Melbourne, 3000 Australia; 11 Illawarra Institute for Mental Health, School of Psychology, Faculty of Social Sciences, University of Wollongong, Northfields Avenue, Wollongong, 2522 Australia

**Keywords:** Help-seeking, Mental health literacy, Resilience, Wellbeing, Self-determined motivation

## Abstract

**Background:**

There is a recognised need for targeted community-wide mental health strategies and interventions aimed specifically at prevention and early intervention in promoting mental health. Young males are a high need group who hold particularly negative attitudes towards mental health services, and these views are detrimental for early intervention and help-seeking. Organised sports provide a promising context to deliver community-wide mental health strategies and interventions to adolescent males. The aim of the Ahead of the Game program is to test the effectiveness of a multi-component, community-sport based program targeting prevention, promotion and early intervention for mental health among adolescent males.

**Methods:**

The Ahead of the Game program will be implemented within a sample drawn from community sporting clubs and evaluated using a sample drawn from a matched control community. Four programs are proposed, including two targeting adolescents, one for parents, and one for sports coaches. One adolescent program aims to increase mental health literacy, intentions to seek and/or provide help for mental health, and to decrease stigmatising attitudes. The second adolescent program aims to increase resilience. The goal of the parent program is to increase parental mental health literacy and confidence to provide help. The coach program is intended to increase coaches’ supportive behaviours (e.g., autonomy supportive behaviours), and in turn facilitate high-quality motivation and wellbeing among adolescents. Programs will be complemented by a messaging campaign aimed at adolescents to enhance mental health literacy. The effects of the program on adolescent males’ psychological distress and wellbeing will also be explored.

**Discussion:**

Organised sports represent a potentially engaging avenue to promote mental health and prevent the onset of mental health problems among adolescent males. The community-based design, with samples drawn from an intervention and a matched control community, enables evaluation of adolescent males’ incremental mental health literacy, help-seeking intentions, stigmatising attitudes, motivation, and resilience impacts from the multi-level, multi-component Ahead of the Game program. Notable risks to the study include self-selection bias, the non-randomised design, and the translational nature of the program. However, strengths include extensive community input, as well as the multi-level and multi-component design.

**Trial registration:**

Australian New Zealand Clinical Trials Registry ACTRN12617000709347. Date registered 17 May 2017. Retrospectively registered.

## Background

Mental disorders are recognised as one of the most prominent contributors to the global burden of disease among young people [1], and carry significant personal, social, and economic costs that can last a lifetime. Half of all mental disorders have their onset before the age of 14 years [2], and young men and boys represent the group at highest risk of mental disorders and suicide in one third of developed countries, including Australia [3]. According to the Australian National Survey of Mental Health and Well-being, 14% of all adolescents aged 12 to 17 years have a mental health issue, with males (16%) showing slightly higher rates compared to females (13%) [4]. The cost to the Australian Government that is associated with mental health problems is estimated to be over $10 billion each year [5].

Australia’s national mental health plan articulates several key outcomes to reduce the national burden of mental disorders. These include, but are not limited to: better understanding and recognition in the community about factors which underpin resilience and prevention of mental health problems; better understanding and recognition of the signs and symptoms of mental health problems; support and assisted help-seeking for early intervention; and, community access to evidence-based treatments and service delivery options [6]. One community-wide strategy for the prevention, promotion, and early intervention for mental health is mental health literacy [7]. Mental health literacy refers to one’s knowledge and beliefs about mental disorders and the efficacy of potential actions to benefit one’s own mental health or that of others [7, 8].

Broadly, mental health literacy is constituted by several distinct components [7]. These include knowledge of preventative mental health strategies, capacity to recognise the development and/or existence of signs and symptoms of mental health disorders, knowledge of effective self-help strategies, capacity to help others who may be experiencing and/or developing a mental health disorder, and knowledge about effective professional help-seeking and treatment options. Importantly, evidence exists to suggest that mental health literacy can be increased at a community level through visible campaigns [7, 9], and that increases in mental health literacy may lead to therapeutic benefits [10]. Furthermore, mental health literacy may be especially beneficial when supplemented with more proactive preventative approaches to mental health such as those encapsulated by the positive youth development movement [11]. As such, a comprehensive approach to community mental health and wellbeing, particularly among young people, is suggested as best approached by dual foci on the explicit and intentional promotion of protective factors in addition to harm-reduction strategies.

There is a recognised need for targeted community-wide interventions aimed specifically at early prevention and intervention [2, 12]. Preventative programs can provide protection against the onset of mental health problems, and specifically, preventative programs aimed at adolescents can reduce and prevent the detrimental long term impact of adolescent mental disorders which reduce the likelihood of completing school, gaining employment, and engaging as a productive member of society [13]. Further, the need for mental health literacy among adolescents and their social support systems is substantial because a large proportion of mental disorders have their onset during adolescence [2] and most adolescents do not have the knowledge or experience to deal with the onset of mental disorders. Young people who recognise a mental disorder are more likely to hold adaptive preferences for help-seeking [14]; however, young males have particularly negative attitudes towards mental health treatment [15] and lack the maturity and experience to deal with mental disorders among their peers despite the fact that peers are often their first option for help-seeking [16].

One particularly motivating and potentially efficacious context for community-wide interventions among adolescent males are organised sports, which play a central role in the Australian lifestyle and national identity. More than two thirds of all Australian boys and adolescent males participate in organised sports each year [17], and they spend on average over 8.5 h each week in participation [18]. Such national prominence and high exposure make organised sports a valuable medium to facilitate population level change in health and health behaviours. For example, a recent Australian study found that prominent sportsmen who publicly disclosed their mental health issues had a positive influence on men’s intentions to seek help and helped to establish help-seeking as a social norm [19]. Further, participation in organised sports during adolescence is associated with a 10–20% reduction in risk for mental health problems when compared with those who drop out of sports [20], while sports participation is also associated with a 29% reduction in suicidal ideation and a 31% reduction in suicide attempts mongst adolescent males [21].

The overall goal of the Ahead of the Game program is to test a comprehensive multi-level, multi-component intervention delivered in community sporting clubs with three primary aims: (1) increase mental health literacy among adolescents and their social support systems; (2) increase help-seeking intentions and attitudes among adolescent male sport participants; and, (3) increase resilience and factors which prevent the onset on mental health problems, including wellbeing and self-determined motivation.

### Aims and hypotheses

The primary research questions are as follows:

1. What effect does the intervention have on adolescent mental health literacy, intentions to seek and provide help, and stigmatising attitudes?
2. What effect does the intervention have on adolescent resilience?
3. What effect does the intervention have on adolescent self-determined motivation?
4. What effect does the intervention have on parents’ mental health literacy and confidence to seek help?

We hypothesise that, compared with adolescents in a control community, adolescents who participate in the intervention will show increases in mental health literacy, intentions to seek and provide help, and a decrease in stigmatising attitudes. We also hypothesise that adolescents who participate in the intervention will show increases in resilience, while those whose coaches participate will also show increases in perceived need support and self-determined forms of motivation. We hypothesise that parents who participate will show increases in mental health literacy. Furthermore, we expect that any potential intervention effects will be moderated by baseline mental health and wellbeing, with greater effects for those who report lower levels of mental health and wellbeing at baseline.

## Methods and design

### Conceptual framework

The Community-Based Participatory Research (CBPR) framework [22] is the conceptual framework for this project. CBPR is a collaborative approach to research that equitably involves all partners in the research process and recognises the unique strengths that each brings. CBPR begins with a research topic of importance to the community (such as the promotion of male mental health) and aims to combine knowledge with action in achieving social change to improve health outcomes and health disparities. The intent of CBPR is for researchers to work side by side with community members to define the research methods, implement the research, disseminate the findings and apply them. Community members become part of the research team and researchers become engaged in the activities of the community, which strengthens the sustainability of public health interventions. Recently, the CBPR framework has become central to the prevention research agenda in the United States, including uptake by the Institute of Medicine and the Centers for the Disease Control and Prevention. This uptake reflects its demonstrated ability to address the complex community issues that public health programs target, triangulation across qualitative and quantitative research methods, and the need to translate findings from basic, interventional, and applied research into changes in practice and policy. We will follow the basic steps of CBPR as detailed by Viswanathan et al. [23].

### Design of the Study

In line with the CBPR framework we will test the comprehensive multi-level, multi-component intervention in organised sporting clubs at the community level. For the purposes of this project we define a community as a group of sporting clubs who are tied together by a singular governing body, who are located close to each other within geographical boundaries defined by the governing sports association, and who compete and interact with one another on a regular (usually weekly) basis. We purposively select the intervention community (a regional area in Eastern Australia) based on a high level of consistency in the predefined boundaries across sports and location to the administering organisation. We match a control community (another regional area in Eastern Australia) based on its similarity to the intervention community in relation to location, size, socioeconomic status, number of sporting clubs, and consistency in predefined boundaries across all participating sports. All sporting clubs from the six most popular sporting codes for adolescent males (Soccer, Australian Rules Football, Cricket, Tennis, Basketball, Swimming; [17]) within each community are invited to participate, enabling a clustered, matched control design to be used for the case control component of the study. Adolescent males in each of the intervention and control communities will be clustered within teams, clubs, and sports. Measures will be taken at the start and at the end of the sporting season (approximately 4 months apart), with participation in the program occurring during the sporting season.

Research undertaken using a nationally representative sample of Australian children has shown that to influence behaviour within the organised sports context it is necessary to intervene at multiple levels of influence and allow for the interplay of influences at multiple levels [24]. Multi-level approaches have been promoted as the gold-standard in community-based health promotion (e.g., [25]) and have established efficacy in promoting change in health behaviours at a community level (e.g., [26]. Multi-level approaches also allow for intervention approaches to be tailored to a detailed assessment of the context in which the intervention is to take place [27]. Based on the socio-ecological model of health promotion [28], we have designed a multi-level intervention specifically targeted at intrapersonal, interpersonal, organisational, and community levels. Further, given the staged roll-out of the intervention over two distinct sporting seasons (i.e., summer and winter), and in keeping with our community-based design, we plan to employ an adaptive design whereby data from season 1 is used to decide if and how improvement to the intervention design can be made for season 2.

### Intervention components

There are four intervention components and a supplementary messaging campaign. Participants will be invited to participate in all relevant programs, but will be able to choose which programs they attend. Attendance at any given program is not dependent upon attendance of another.

### “Help out a mate”: A brief mental health literacy program for adolescent male athletes

During formative research focus groups, adolescent males asked for more information about mental health problems, how to recognise them, and how they can help out their friends if they are experiencing a mental health problem. It has also been found that some of the most common reasons that young people who experience mental health problems do not seek professional help are high levels of stigma and poor mental health literacy [4]. Therefore, a brief sports-based mental health literacy program could meet these specific needs. Although adolescents prefer to disclose problems to their friends [29], adolescent peers may not be appropriately equipped to deal with problems when they are called upon for support [30–32]. The Help Out a Mate intervention aims to increase mental health literacy among adolescent males (specifically in regard to depression and anxiety), increase their skills and confidence to help a peer showing signs of a mental health problem, increase helping behaviour and supportive actions, increase appropriate help-seeking among young males at risk of mental health problems, and decrease stigmatising attitudes.

The intervention addresses the components of mental health literacy as articulated by Jorm [7], with the content and structure developed on review of literature and existing programs and materials [33–36]. The program is designed for the sport context (e.g., being a good team mate) and is deliberately brief (approx. 45 min) so that it can conveniently be placed in or around a typical training/practice session. Specifically, the intervention involves the following components; (i) what is mental health and mental illness; (ii) myths about mental illness; (iii) what is depression?; (iv) what is anxiety?; (v) how to provide help; and, (vi) where to get reliable information. The program focuses on helping adolescents to recognise the signs of depression and anxiety, approach a friend confidently, encouraging help-seeking, make an adult aware of potential mental health problems, and understanding self-help behaviours. Additionally, the program addresses the issue of how to ask for help if an adolescent feels that they need it. Additional resources provided to adolescents, include a ‘Man Card’ (business card), which lists key steps on how to help a friend, and a list of mental health resources.

The program is to be delivered once using a face-to-face approach and where possible it will be conducted in a room at the athlete’s sports clubs to groups of up to a maximum of 30 adolescents (approximately two teams). The program will be facilitated by trained volunteers, for whom it is desirable that most have lived experience of mental health problems, as it has been found that a mixture of education and exposure to someone with a mental illness can effectively reduce stigma [37]. All volunteers will also be accredited in Mental Health First Aid [36]. The hypothesised outcomes of the program are increases in literacy for depression and anxiety, increases in intentions to seek help, increased confidence to provide help, and decreases in stigmatising attitudes.

### Your path to success in sport: An internet-supported resilience intervention for adolescent males

This intervention aims to increase psychological resilience: mental processes and behaviour which promote personal assets and protect an individual from the potential negative effect of stressors [38]. Specifically, this intervention will target key psychological skills identified through a review of sport-based resilience literature (e.g., [38–42] aimed at helping adolescent males to cope with the inevitable adversities of life through explicit sport-based examples. The program is delivered via brief workshop (approx. 45 min) supported by six internet-based (website/mobile application) modules.

The face-to-face workshop is delivered once, and conducted, when possible, in a room at the athlete’s sports clubs to groups of up to 30 adolescents (approximately two teams). The workshop is facilitated by trained presenters who have been educated in psychology to at least undergraduate level. The workshop is framed around expectations vs reality in the process of achieving goals (i.e., the “path to success”) to identify inevitable challenges, obstacles, and adversity that adolescents are likely to face in and outside of sport. Then, the workshop addresses the need to build core skills through which the adolescents can overcome adversity, relating directly to the internet-based resources, which are promoted. Videos and interactive activities support the presentation.

The internet-based component of the intervention is delivered via website and/or mobile phone application. The website is accessible on any internet-enabled device, and the app is available to download on mobile devices. Each of the six core modules takes approximately 10–15 min to complete, and they are designed to be completed in order (i.e., the first module had to be completed before the second was unlocked). Informed by sport-based resilience literature (see above), the modules are: (i) problem-solving; (ii) controlling the controllables; (iii) managing your thoughts; (iv) keeping your cool; (v) playing to your strengths; and (vi) appreciating your team. An additional recap module was also available to participants who completed all six preceding modules (e.g., with summary material and evaluative questions). Participants will be able to complete all modules in one session if desired, however the content is designed so that participants can continue to engage with and practice the exercises after all modules were completed. These skills are delivered through informational videos, infographics, reflective activities, and exercises to try at the end of each module. This component of the intervention will be delivered individually, and participants are required to log in to access the materials. The hypothesised outcomes of the program are more adaptive implicit beliefs about managing adversity, and increases in resilience.

### Parent program: Mental health literacy

Parents are the primary source of support for adolescents (Jorm & Wright, 2007) and likely one of the first observers of mental health disorder symptoms in their children [43]. Therefore, parents need to be able to provide adequate support and assistance when their child or adolescent shows symptoms of a mental health disorder [44]. Research, however, suggests that the mental health literacy of parents (in terms of their children’s mental health) is limited [45] and parents are not adequately prepared or confident to assist children who experience a mental health disorder [46].

The parent program is designed to increase parent mental health literacy through a one hour, face-to-face workshop delivered through community sport clubs. Specifically, the workshop aims to raise awareness of parents’ role in promoting and supporting positive adolescent mental health, and to increase knowledge of common youth mental health disorders, mental health-promoting behaviours, and help-seeking options. It also aims to reduce stigma, promote constructive communication about mental health, and increase parental confidence and self-efficacy in supporting adolescent mental health. The design, content, and delivery of the intervention has been informed by recent qualitative work that researched parental perceptions of mental health literacy interventions and their potential use in youth sport settings [47].

Intervention content is guided by a mental health literacy framework [48] and is designed to be engaging through a mix of parent reflection, discussion, presentation, and videos. Materials developed and adapted were either from Mental Health First Aid guidelines [49, 50], or are used with permission from mental health organisations and parenting organisations (e.g., ReachOut, Raising Children Network). The workshop is kept intentionally brief to combat parents’ reported time constraints, and capitalises on the close social support networks among parents in the youth sport club environment [47]. The information presented is set at an introductory level with supplementary online material offered via the intervention project website (aheadofthegame.org.au).

### ABCs of motivation: Internet-supported coach education program

We based the content for this component on an intervention previously shown to reduce mental ill-being (i.e., athlete burnout) in male adolescent sport participants [51]. Using self-determination theory as a guiding framework [52], we designed the intervention to teach coaches strategies to support their players’ basic psychological needs, including autonomy (feeling self-directed and capable of making choices about one’s actions), competence (feeling effective in one’s interactions with the physical and social environment), and belongingness (feeling closely connected and cared for by others).

Langan and colleagues’ [51] original program involved six face-to-face training sessions delivered by a sport psychologist to each coach, on a one-to-one basis. In the current study, we adapted the intervention so that it could be delivered more widely than in the original study. Formative research for the current project indicated that youth sport coaches believed that some aspects of the program could be delivered using online resources, but they also believed that it would be important to meet with facilitators in order to ask questions and discuss specific implementation challenges. To address these concerns, we designed a blended delivery model, involving face-to-face workshops, online self-paced tasks, online mentoring (via Google ‘Hangouts’ video-based platform) and face-to-face mentoring in small groups (e.g., five coaches, one mentor). A registered sport psychologist will facilitate all workshops and mentoring sessions. Coaches will complete online tasks using a content management system designed for the project. See Table 1 for details.

**Table 1:** Sample overview of coach training

Modules	Mode	Timing
1. Coach Control – Helpful or Harmful?	Workshop 1 (2 h)	Week 1
2. Feedback – ‘What’ You Say and ‘How’ You Say it Matter		
3. The Importance of Having a Plan – and Ensuring Your Athletes Understand it	Online 1 (30 min)	Week 2
4. Rationale – The Importance of ‘Why’	Online 2 (30 min)	Week 4
5. Building Athlete Togetherness	Online 3 (30 min)	Week 6
	Mentor Session 1 (1 h)	
6. Athlete Choice – Why, What, & When	Workshop 2 (2 h)	Week 8
7. Developing Independent Athletes – The Skill of Standing Back		
8. Building Long-Term Motivation - Defining Athlete Success		
9. Building Great Relationships with Your Athletes	Online 4 (30 min)	Week 10
10. Reacting to Resentment – and Other Negative Player Emotions	Online 5 (30 min)	Week 12
11. Ensuring Sport Skills Build Life Skills	Online 6 (30 min)	Week 14
	Mentor Session 2 (1 h)	

### Mental health messaging campaign

To support the Ahead of the Game program, a club-based promotional campaign was developed to improve mental health literacy to increase knowledge and attitudes about mental health and encourage help seeking in adolescent males (both for self and to help others). To inform, design and develop the campaign, a social marketing approach was utilised and included formative research, message development and concept pre-testing. Social marketing has been used successfully in a variety of health promotion interventions to increase awareness of health issues and promote behavior change [53]. In line with the CPBR framework, this requires meaningful involvement with the target audience to develop and design the intervention to ensure understanding of the relevant issue and associated behaviours. Thus the messages, concepts and promotional aspects for this campaign were developed based on the key insights from focus groups with adolescent males in the Illawarra. This included focusing on mental health to encourage help seeking with an emphasis on how to help others; including emotional and physical wellbeing to be consistent with the other components of the overall program; focusing on prevention and noting that sport/physical exercise impacts on both mental and physical wellbeing; addressing stigma around manliness, including showing or talking about feelings and emotions as well as beliefs about mental health issues resolving on their own without relevant help.

Three concepts were pre-tested to elicit views and understanding on images, taglines and messages. Based on the analysis, the final ‘Man Card’ concept (e.g., You won’t lose your Man Card for getting help) was chosen as the audience felt this would best resonate with their peers due to the use of humor, images being credible and relatable, but most importantly they felt that it had relevant and positive mental health messages. Final materials including posters, banners, branded merchandise and a campaign specific website will be disseminated across sports clubs in the Illawarra region in 2016/2017 to support the adolescent programs outlined previously.

### Control group

Participating clubs in the control group will not receive any program during the study period, but will be offered a mental health literacy program upon completion of the study. The INSIGHTS program is a community presentation aimed at teenagers and primarily delivered at secondary schools around Australia [Black Dog Institute, 2014. Accessed 01/10/2017. Available at: https://www.blackdoginstitute.org.au/education-training/community-and-schools/free-school-presentations/insights]. The presentations typically last an hour and are delivered by trained volunteers with personal experiences with mood disorders, who weave aspects of their story into the material presented. The presentation uses humorous illustrations to help explain concepts that young people find difficult to articulate and the topics covered include: pressures faced by teenagers today; signs and symptoms of depression and bipolar disorder in young people; identification of early warning signs; when and where to seek help; practical strategies for offering support; and strategies for building resilience.

### Measures

To measure mental health literacy, 13 items of the Depression Literacy Questionnaire [54], 13 items of the Anxiety Literacy Questionnaire [55], and one item from the Mental Health Literacy Scale, “I am confident that I know where to seek information about mental illness” [56] will be used. To measure help-seeking intentions, the General Help Seeking Questionnaire will be used [57] and to measure stigmatising attitudes, the Social Distance Scale will be used [58]. Items from the Mental Health Literacy Survey will also be included to investigate experiences with mental health, mental health first aid behaviours, and confidence to provide help [59]. If participants indicate that they have had contact with someone they believed to have a mental health problem, they will be asked if they tried to help, and are also asked to indicate what they did to try and help. In order to measure Mental Health First Aid intentions, participants will be asked to rate on a seven-point scale how likely they would be to do a list of five behaviours that is drawn from the content of the program. Items have been adapted from a previous measure [34]. Adolescent resilience will be measured using the 10-item version of the Connor-Davison Resilience Scale [60]. Implicit beliefs regarding one’s ability to deal with problems will be measured using three items adapted from previous research [61]. Perceived parental social support will be measured using the four items of the parental support subscale of Multidimensional Scale of Perceived Social Support [62]. In addition, parents’ are asked to report on 15 items of the Mental Health Literacy Scale (O’Connor & Casey, 2015).

To measure the effects of the coach education program we will use measures of athlete engagement, athlete burnout, perceived needs support, coach controlling behaviour, basic needs satisfaction, and self-determined motivation. Athlete engagement will be measured using the mean of four items measuring confidence, vigour, dedication and enthusiasm drawn from the Athlete Engagement Questionnaire [63]. Athlete burnout will be measured using the mean of three items assessing emotional and physical exhaustion, devaluation, and reduced sense of accomplishment drawn from the Athlete Burnout Questionnaire [64]. Perceived needs support will be measured using the mean of three items assessing autonomy, competence and relatedness drawn from the Perception of Needs Support Scale [51]. Controlling coach behaviour will be measured using the mean of four items drawn from the Controlling Coach Behaviour Scale [65] measuring controlling use of rewards, negative conditional regard, intimidation, and excessive personal control. Satisfaction of basic needs will be measured using five items drawn from the Basic Need Satisfaction in Sport Scale [66]. Three items will be used to measure autonomy, and one item each to measure competence and relatedness. Self-determined motivation will be measured using five items drawn from the Behavioural Regulation in Sport Questionnaire [67], with items measuring amotivation, external regulation, introjected regulation, identified regulation, and intrinsic motivation.

To explore potential effects of the program on adolescent males’ mental health and wellbeing, two measures are used. Psychological distress is measured using the Kessler-6 (K6); [68], a six-item questionnaire assessing the level of depressive and anxiety symptoms that have been experienced within the past month. Wellbeing is measured using the short form of Keyes’ Mental Health Continuum (MHC); [69]. This is a 14-item measure of an individual’s emotional, psychological and social wellbeing. The short form of the MHC has shown excellent internal consistency (> .80) and discriminant validity in adolescents. All measures will be taken at three time points: (i) prior to commencement of participation in Ahead of the Game programs, and as close to the start of the sporting season as possible; (ii) immediately following participation (i.e., post workshop); and (iii) six weeks after last involvement with Ahead of the Game, or immediately prior to the end of the sporting season, whichever is longer.

To supplement quantitative measures, qualitative evaluation of all intervention components is planned to obtain a richly textured, detailed understanding of the project from the perspective of participants. This qualitative component moves beyond the measures chosen by researchers to provide stakeholders with an opportunity to express their own perceptions and experiences of the programs. Specifically, focus groups and interviews are employed to understand the perceptions and experiences of adolescents, coaches, and parents regarding intervention programs. This component of the evaluation is expected to generate new insights into ‘what works’ and ‘what doesn’t work’ from the perspectives of those who have undertaken the program.

### Recruitment and engagement strategy

The project engagement strategy is informed by the guiding principles outlined in the Stakeholder Engagement Framework (e.g. reciprocity, inclusion, transparency, and respect) [70], in keeping with our CBPR priorities. The engagement strategy has three phases. Phase 1 – Building awareness: Key messages are developed concerning the need for and timeliness of the project, as well as the involvement of the Illawarra community in developing Ahead of the Game interventions. These particularly emphasise gains for the club and the mutually beneficial nature of involvement for all stakeholders [71]. Public awareness of the project is through disseminating key messages primarily with local and online media in the first instance, followed by dissemination via the project website, social media accounts, community presentations, and leveraging the capacity of partner organisations. Phase 2 – creating relationships with key stakeholders: Strategic partnership opportunities will be identified by scoping the organisations and/or peak bodies at national, state and local levels with the capacity to connect Ahead of the Game directly with local clubs across the six sports. The research team directly approach identified organisations to gain their support and ascertain their willingness to facilitate recruitment. Where possible, opportunities for identifying ambassadors, attending club meetings and generating media coverage are explored. All partner organisations are then listed on the official project website. Phase 3 – Recruitment of community clubs: Building on public awareness of the project generated in Phase 1, all sports clubs in the Illawarra and Central Coast regions are invited to participate in the Ahead of the Game trial. Clubs are notified about the project prior to the season starting, firstly by email/newsletter contact from governing bodies and invited presentations at all-clubs meetings, and secondly, in writing (email or letter directed to the club president) by the research team. Clubs will be offered individual club presentations on an as needed basis. Additionally, individuals will be able to fill in an expression of interest via the website.

To maximise consent rates, club engagement officers will be employed to liaise and follow up with all invited clubs, using scripted telephone calls and engagement checklists. At the point of recruitment club representatives will sign a letter of support indicating their commitment to implementing the program and appoint a club-level ‘champion’ for the duration of the project. Throughout the engagement process, the team will document stakeholders’ motivation to engage or disengage with the goals of the project.

### Sampling and recruitment of participants and stakeholders for qualitative evaluation

Interviews and focus groups will take place with purposively sampled participants and sporting organisation stakeholders (e.g., club presidents, player development coordinators) approximately a month after the final questionnaire. Adolescent participants will be recruited through their teams/groups (i.e., for focus groups), while parents and coaches will be recruited individually (i.e., for interviews). All individuals who participated in the program will be invited to take part in interviews or focus groups, regardless of the extent to which they completed the program (in order to facilitate a maximum variation sample). Those selected for interviews/focus groups will be sent an additional participant information sheet and consent form by the research team. Data will be collected until saturation is reached. The interviews and focus groups will explore acceptability of the intervention and research design, perceived impact of the intervention, and strategies for refinement and dissemination.

### Implementation strategy

We will aim to engage in a scientifically grounded implementation strategy for this multi-setting, multicomponent, multi-level intervention. As with most mental health interventions, successful implementation requires an interaction among multiple individuals, organisations, funders, and systems to be successful, thus making interventions highly complex to implement. The aim of the implementation strategy is to effectively promote adoption of intervention components. Implementation of evidence-based and evidence-informed practices in mental health is critical for improving health outcomes. The strategy is informed by two implementation frameworks– the National Implementation Research Network (NIRN) model of implementation and the Consolidated Framework for Advancing Implementation Research (CFIR) [72, 73]. These conceptual models are complementary; the NIRN model [73] characterises the overarching staged processes of implementation and key drivers for successful implementation, while the CFIR model explores the role of various constructs at multiple levels of influence [72].

### Sample size calculations

To calculate the required sample size we have used the general indicator of psychological distress (K6) rather than other program specific outcomes. Based on pilot testing using two independent samples with a between group difference of 1.2 (*SD* = 4.0), adjusting for clustering using a design effect of 1.35 it is estimated that 231 subjects are required in each group (with an alpha of 0.05 and 80% power).

### Qualitative evaluation sample size

The qualitative evaluation will collect data until saturation is reached. We anticipate that up to 50 adolescent participants, 20 parents, and 10 coaches will be included.

### Data analysis

#### Analyses of primary and secondary outcomes

Between-group differences on the primary and secondary outcomes will be analysed according to both intention-to-treat and per-protocol principles, using appropriate multivariable statistics (linear mixed models, generalised linear models adjusting for clustering and covariates such as age and socioeconomic status).

#### Mediation and moderation analyses

Two analyses will be conducted to explore potential mediators and moderators of the intervention effects. First, hypothesised mediators of change in primary outcomes (e.g., mental health literacy) will be examined. Potential moderators of the intervention effects (e.g., socioeconomic status, age, and geographic location) will also be explored using multi-level modelling.

#### Per-protocol analyses

A per-protocol or dose-response analysis will also be performed at the club and individual levels. Adolescents’ individual-level compliance will be measured using the following indicators: having a coach who completes the coach training program; a club who implements the messaging campaign; at least one parent who has received the parent program; attendance at the Help Out a Mate program; and, attendance at the Your Path to Success in Sport program. All participants and clubs will be included in the intention-to-treat analyses.

#### Qualitative analyses

All interviews and focus groups will be digitally recorded and transcribed verbatim. Data will be analysed thematically (e.g., [74]), and strategies will be employed to enhance rigour and trustworthiness [75].

## Discussion

This study utilises a community-based participatory research framework to test a multi-level, multi-component intervention designed to enhance mental health and wellbeing and reduce the risk of the mental health problems among adolescent males who participate in organised sports. The intervention will be implemented at a community level and will be tested against a matched case control community. Specifically, the intervention targets adolescents, as well as their peers (teams), parents, coaches, and the sporting environment by embedding four programs and a complimentary messaging program within community sporting clubs. The dual foci of the program are the explicit and intentional promotion of protective factors in addition to harm-reduction strategies. Protective factors include high levels of psychological well-being, high levels of social support, and self-determined forms of motivation. Early intervention and harm reduction strategies include high levels of mental health literacy, intentions to seek and provide help for emerging mental health problems, and knowledge of options for professional help.

Organised sports are a promising avenue for mental health promotion, particularly among adolescent males. High participation rates and prolonged engagement in organised sports make them conducive to the delivery of programs such as Ahead of the Game [17, 18]. In considering study limitations we note that intervening in a sample of sports participants presents a risk of bias to the study given that sports participation is associated with better mental health and non-participation is associated with higher risk for mental health problems [20]. As such, those who are most in need of intervention may not be systematically exposed to any program which is implemented solely through community sporting clubs. Furthermore, the multi-level design could be expected to lead to greater change in primary outcomes, as well the maintenance of potential benefits. However, the multi-level design may enhance a form of self-selection bias whereby adolescents with strong social support systems may be more likely to participate and more likely to have coaches, parents, and peers who also participate. This is particularly relevant for per-protocol analyses.

Our community-based approach has notable strengths. For example, to achieve meaningful and sustained benefits, stigma needs to be redressed where it is structurally embedded within our societies [76] and a community-based approach to stigma-reduction can leverage the cumulative reach of individuals within the contexts in which they interact. Furthermore, community involvement in the design and delivery of the Ahead of the Game programs may lead to higher levels of community ownership and engagement during the implementation phase [72]. However, the matched control design increases the risk of bias from potential confounding effects such as school-based initiatives being run within the selected communities. While this is inherent in the case control design, triangulation of case control findings with pre-post analysis from the intervention group is expected to mitigate this risk.

One significant risk to the fidelity of implementation is the translational nature of the program, as well as the focus on health promotion and prevention in a complex community setting [77, 78]. As such, the study is more akin to an effectiveness, rather than an efficacy study, whereby the ‘real life’ benefit derived from ‘real life’ programs is examined [79]. In this respect, there are potentially several factors which may impede successful uptake and implementation, and therefore the effectiveness of the program. These include the part-time or volunteer nature of those who run community sporting clubs, an unknown level of acceptability and community ownership of the program and its potential consequences among target clubs, and high levels of research related tasks such as survey instruments which demand significant investments of time. To try to overcome the potential risks to effectiveness, we have articulated an implementation plan based on the NIRN and CFIR models [72, 73], as described in detail above. However, there is a dearth of evidence to suggest which constructs should be prioritised in a community sport sample. Through the qualitative evaluation we hope to be able to contribute to the knowledge around optimising community ownership and implementation of health programs within community sporting clubs. Furthermore, we also hope to provide data on the feasibility and uptake of all intervention components within the community.

## Conclusion

Organised sports are one potentially engaging avenue through which to promote mental health and prevent the onset of mental health problems among adolescent males. The community-based design with samples drawn from an intervention and matched control community allows an evaluation of the effectiveness of the multi-level, multi-component Ahead of the Game program on adolescent males’ mental health literacy, help-seeking intentions, stigmatising attitudes, motivation, and resilience. Effects on psychological distress and wellbeing will also be explored. The community based design presents several challenges to successful implementation which have been planned for through the use of evidence-based implementation models.

## Abbreviations

CBPR: Community-Based Participatory Research
CFIR: Consolidated Framework for Implementation Research
K6: Kessler-6
MHC: Mental Health Continuum
NIRN: National Implementation Research Network
